# Microbiome analysis reveals microecological advantages of emerging ditchless rice-crayfish co-culture mode

**DOI:** 10.3389/fmicb.2022.892026

**Published:** 2022-07-22

**Authors:** Xiaoli Huang, Minghao Li, Ya Huang, Hai Yang, Yi Geng, Ping Ouyang, Defang Chen, Lizi Yin, Shiyong Yang, Jun Jiang, Wei Luo, Zhi He

**Affiliations:** ^1^Department of Aquaculture, College of Animal Science and Technology, Sichuan Agricultural University, Chengdu, China; ^2^Haide Aquatic Technology Co., Ltd, Yibin, China; ^3^College of Veterinary Medicine, Sichuan Agricultural University, Chengdu, China

**Keywords:** aquaculture environment, ditchless rice-crayfish cultivation, gut microbiota, *Procambarus clarkii*, bacterial interaction

## Abstract

Ditchless rice-crayfish co-culture is an emerging model of rice-crayfish farming that circumvents the potential hazards of digging ditches in traditional rice-crayfish farming. However, due to the complex interactions among crayfish, ambient microbiota, and environmental variables, it is necessary to assess the differences in bacterial structure between ditchless and traditional rice-crayfish culture. In this study, the crayfish culture area in the Sichuan basin was selected as the study area, and the bacterial communities of two rice-crayfish culture systems were compared by high-throughput sequencing of 16S rDNA. The results showed that the ditchless system had lower water depth, higher dissolved oxygen, lower total ammonia nitrogen and lower morbidity. There are intuitive differences in the composition of environmental bacterial communities due to environmental changes, even if they are similar in composition at the phylum level. Microbiota in sediments from ditchless systems appear to produce less ammonia nitrogen. The abundance of the pathogens colonizing the intestine of ditchless crayfish was lower than ditched one, and the composition was similar to water. Ditch-farmed crayfish appear to be more susceptible to environmental microbes and have a more fragile intestinal structure. Water depth and dissolved oxygen are the main environmental factors that determine the distribution of microbiota. This study is the first to investigate the bacterial ecology of a ditchless rice- crayfish farming system. The results show that the ditchless rice-crayfish culture model has a more superior bacterial system than the traditional rice-crayfish culture.

## Introduction

The crayfish (*Procambarus clarkii*), a member of the Cambaridae family, was introduced to China in the 1930s and has become popular for its delicious meat (Li et al., 2012). In recent years, rice-crayfish co-culture, a revolutionary farming model, has been widely promoted in China, with 12,613 km^2^ of farming area and an annual production of 2.06 million tons of crayfish, accounting for 86% of the total production (MARA, 2021). Compared to traditional pond farming, rice-crayfish co-culture systems take advantage of the shallow water environment and the idle production period of the paddy fields in winter, and also increase the utilization and productivity of the fields (Si et al., 2017). However, the traditional rice-crayfish model requires digging ditches around the paddy fields. In Sichuan Province, for example, the depth and width of the ditches are required to be 150 to 200 cm and 200 to 300 cm, respectively, covering about 10% of the entire paddy area, to provide living and breeding sites for crayfish (Affairs, 2021). From the production practice in the past decade, ditches have reduced the area and the yield of rice cultivation (Hu et al., 2016). Since ditches occupy a large area of paddy fields, they are not suitable for promotion in hilly areas with little land resources and narrow areas, which leads to the concentration of farming areas mainly in plain regions (Hou et al., 2021). Furthermore, deposition of feed residues and aquatic animal excreta may also lead to environmental stress in ditch-farmed rice-crayfish co-culture systems, resulting in high mortality during the hot season (Cao et al., 2017; Shen et al., 2020). Due to the problems posed by ditches, an emerging model of farming, ditchless rice-crayfish co-culture, has been proposed and implemented in recent years. Since no ditching is required, the rice culture area is increased directly and crayfish have a greater range of movement, avoiding localized water and sediment deterioration and reducing mortality (Huang J. et al., 2020). In addition, compared to traditional rice-crayfish farming systems, ditchless rice-crayfish farming systems increase the area of rice planted by about 10%, thus increasing the economic efficiency. Therefore, the ditchless rice-crayfish farming system has research value as a potential sustainable farming model.

Intestinal microbiota is symbiotic with the host and play an important role in immune response, digestive physiology, and regulation of body functions (Mueller et al., 2012). The intestinal microbiota of aquatic animals are greatly influenced by the environmental bacterial community, which means they may be more susceptible to environmental microbiota (Zhao et al., 2020). On the other hand, the distribution patterns and trait variation of environmental microbiota are influenced by environmental conditions (Chen et al., 2021). Due to the complexity and high variability of aquatic ecosystems in rice fields, environmental microbiota may be more significantly affected by environmental changes. Compared to vertebrates, aquatic invertebrate crustaceans lack typical adaptive immunity, and a stable intestinal microbiota is more important in disease prevention (Cerenius et al., 2010). As benthic organisms, crayfish are between the water and the sediment, and are affected by the microbiota in the sediment and aquaculture water environment. This bacterial environment may have unique regulatory mechanisms on the intestinal microbiota of crayfish and affects the health of the host. Given the important role of intestinal microbiota in regulating the health of aquatic animals and the impact of environmental changes on surrounding microbiota, understanding the ecological patterns of crayfish intestine and environmental microbiota is important for the engineering design of aquaculture (Hou et al., 2020; Huang Z. et al., 2020). However, the bacterial communities of the ambient water, sediment and intestine in ditchless rice-crayfish co-culture systems are still relatively unknown.

In the present study, we aimed to understand the differences in bacterial ecology between ditched and ditchless rice-crayfish co-culture systems. For this purpose, water, sediment and intestine samples were collected from ditch and ditchless systems in Yibin, China, a region with a diverse topography and a preference for rice-crayfish culture. In addition, our findings will help to provide decision makers with informative suggestions regarding the design of culture engineering by comparing bacterial patterns in different aquaculture systems.

## Materials and methods

### Sampling area

**FIGURE 1:**
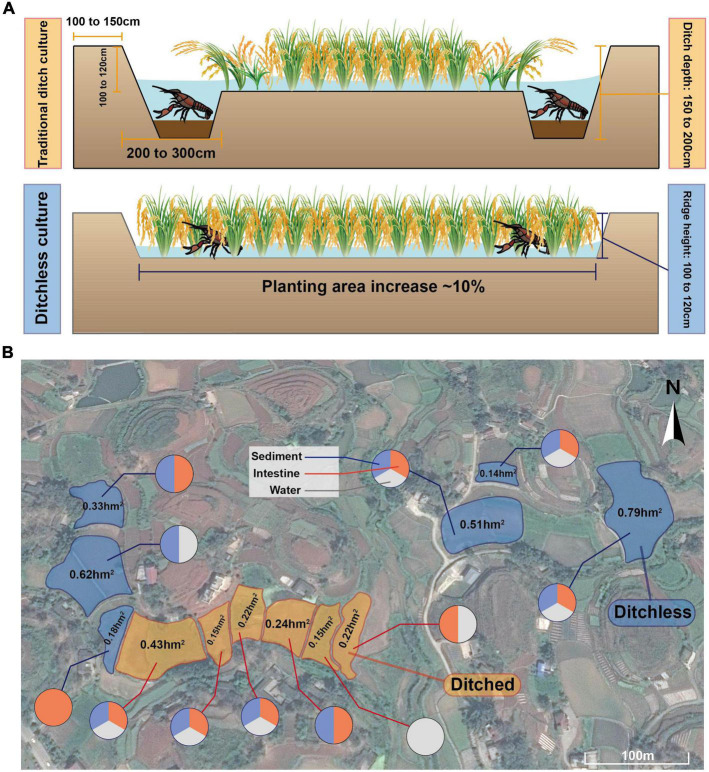
Sample collection site in Yibin, Sichuan, China. **(A)** Schematic diagram of ditched and ditchless rice-shrimp co-culture systems. **(B)** Specific distribution of sampling points and type of samples collected.

The experiment was selected to be conducted in Nanxi District, Yibin City, Sichuan Province, China (104°96′E, 28°87′N), located in a typical mountainous hilly region in the upper Yangtze River. The climate in this region is subtropical humid monsoonal, with an average annual rainfall of 1072.71 mm, an average annual frost-free period of 348 days, and an average annual temperature of 18.7°C, which is suitable for crop growth. Meanwhile, the region has a well-developed aquaculture industry because of its abundant water resources, including crayfish as a special breed with an annual production of more than 1,800 tons. All samples were collected from real farming regions, and the farming regions and the types of samples were shown in Figure 1. The area of the farming region ranged from 0.14 to 0.79 hm2. All farming regions were built on the topography of the hills and irregular in shape. Fertilizers and pesticides were not permitted to use during breeding in both models. The same commercial feed (Purina, Cargill) was fed at 3% of the total crayfish weight every afternoon and the culture water used was from the same source.

### Sample collection

All intestine, water and sediment samples were collected from two different culture model areas that had been utilized for 45 days in March 2021. Each culture model area contains six culture sites. During sampling, the dorsal carapace of crayfish with similar weight was opened and the whole intestine was cut off and rinsed several times with sterile phosphate buffer. Three crayfish intestines were combined into one intestine sample for DNA extraction. Water samples were collected from the area at a depth of 30 cm using the 5-point method, and then 500 mL of the mixture was filtered through a 0.22 μm pore size microfiltration membrane to obtain microbe samples for our analysis. Three surface sediment samples from different points in each area were eventually mixed into a single sample with a wet weight of approximately 500 g to represent the area. To reduce errors in the outdoor data, samples of the same type for each culture mode were mixed to finally obtain three regional replicates. The corresponding label information of samples was shown in Supplementary Table 1. All samples were collected and rapidly frozen with liquid nitrogen and stored in a −80°C refrigerator until DNA extraction.

### DNA extraction, bacterial 16S rRNA gene amplification and miSeq sequencing

The total DNA was extracted from the water, sediment, and intestinal contents using a bacterial DNA isolation kit (Foregene, Chengdu, China) according to the manufacturer’s instructions. The DNA extract was checked on 1% agarose gel, and DNA concentration and purity were determined with NanoDrop 2000 UV-vis spectrophotometer (Thermo Fisher Scientific, Wilmington, CA, United States). The V3–V4 hypervariable region of 16S rRNA was amplified using the 338F (5′-ACTCCTACGGGAGGCAGCAG-3′) and 806R (5′-GGACTACHVGGGTWTCTAAT-3′) primers. The PCR product was detected using 2% agarose gel, purified using the AxyPrep DNA Gel Extraction Kit (Axygen Biosciences, Union City, CA, United States) and quantified using Quantus™ Fluorometer (Promega, United States). High-throughput sequencing was performed in a paired-end model using the Illumina MiSeq PE300 platform (Illumina, San Diego, CA, United States).

### Bioinformatics analysis

After Miseq sequencing, non-conforming reads were removed from the raw data by using Qiime (version 1.9.1). Filtered sequences were clustered into operational taxonomic units (OTUs) at 70% confidence level by using Uparse (version 7.0.1090)^1^ *via* the SILVA rRNA database. The RDP Classifier was used to obtain OTU annotation information at a 97% similarity level. Alpha diversity, including Shannon, Simpson, chao and Coverage index were calculated by using Mothur (version v.1.30.2). Beta diversity and hierarchical clustering trees constructed by the unweighted pair group method with arithmetic mean (UPGMA) were used to compare the similarity of comparative cluster composition. The artificially constructed FAPROTAX database was used to annotate the metabolic or ecologically relevant functions of biological taxa. The functional abundance profiles of bacterial communities in different systems were inferred and obtained from 16S rRNA marker gene sequences by using PICRUSt II. Networkx was used to show the distribution between samples and species and to calculate the species-to-species correlations. Redundancy analysis (RDA) was selected to address the impact of environmental variables on the system microbiota with the vegan package in the R software.

### Determination of water quality

The water depth of the ditched system was defined as the distance from the bottom of the ditch to the water surface, while the ditchless system was the distance from the sediment surface at the bottom of the paddy field to the water surface. Each area was measured using the five-point method. The temperature at 30 cm below the water surface is measured with a thermometer. The dissolved oxygen and the pH in water were measured with the dissolved oxygen meter and pH meter, respectively. Water transparency was assessed by the Behcet’s disk method. The breeding logs for the study area from February 15 to April 15, 2021 were provided by the technicians. All data were recorded to Excel and counted.

### Statistical analysis

All data were expressed as mean ± standard deviation. Significant differences between the two systems were determined using Student’s *t*-test and non-parametric Wilcoxon signed-rank tests. The level of statistical significance was accepted by as *P* < 0.05. Statistical analysis and data visualization were performed on the online platform.^2^

## Results

### Site characteristics

Supplementary Table 2 showed the physicochemical properties of the different sampling sites and the status of crayfish culture. The results showed that ditchless culture has a lower water depth. The higher water temperature and dissolved oxygen in the ditchless culture may be due to the shallower water. Total ammonia nitrogen and nitrite were higher in the ditch system than in the ditchless system. There was no difference in pH and water transparency between the two culture systems. It is interesting to noted that morbidity and mortality were higher in traditional ditched farming compared to ditchless farming, which may implied that environmental differences in different farming systems can affect the farming species.

### Global analysis of 16S rDNA sequencing

To further explore possible differences between the two culture systems, we analyzed the microbiota in 18 samples from 12 different sites (Supplementary Table 1). After quality filtering and assignment, a total of 846,508 high-quality sequences with an average length of 419 bp were obtained. A total of 5,281 OTUs were annotated, belonging to 2,222 species, from 1,139 genera, from 56 phyla. The Shannon and Simpson indices were used to calculate species diversity. The results showed that bacterial diversity was highest in the sediment, intermediate in the water, and lowest in the crayfish intestine (Supplementary Figure 1A and Supplementary Table 1). The Chao index used to calculate community richness showed a similar trend to the diversity analysis (Supplementary Table 1). In addition, the rank-abundance and PAN also illustrated the differences in community richness among different types of samples (Supplementary Figures 1B,C). The coverage of all samples was greater than 97%, and there was a high similarity among the replicates (Supplementary Figures 2, 3).

**FIGURE 2:**
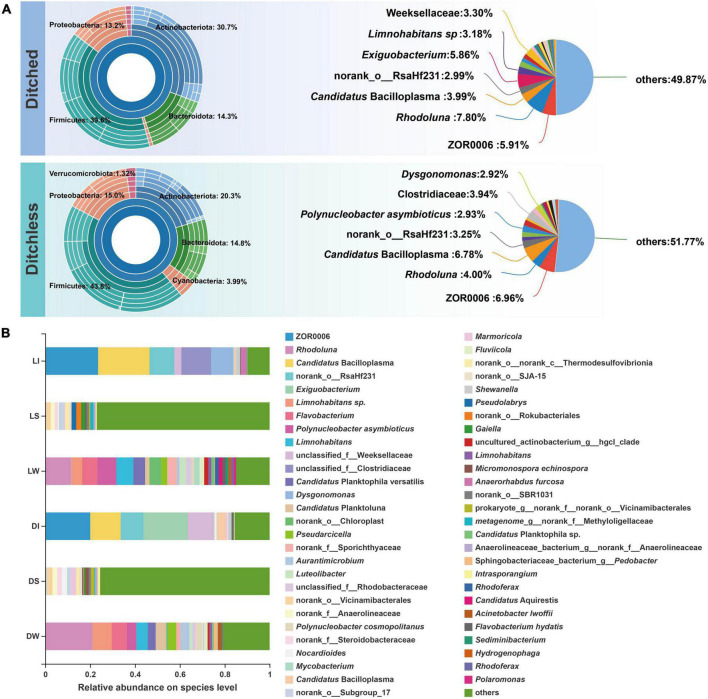
Bacterial community structures of ditched and ditchless systems. **(A)** Sunburst plots and pie charts of the most abundant phyla. The samples of the ditched system include DI, DW, and DS. The samples of the ditchless system include LI, LW, and LS. **(B)** Species composition of each sample compiled from three replicates.

A total of 326 bacterial species were found in all samples. The different types of samples showed similar annotation in both culture systems, the most unique species in the sediment and the least unique species in the crayfish intestine (Supplementary Figure 4A). Furthermore, no significant difference in diversity was found between the two systems (Supplementary Figure 4B). The multilevel species Sunburst diagram (Figure 2A) showed that bacteria of both culture systems belonged to six main phyla, i.e., Firmicutes, Actinobacteriota, Bacteroidota, Proteobacteria, Cyanobacteria, and Verrucomicrobiota. Among them, the ratio of Actinobacteriota and Cyanobacteria were distinctly different between the ditched and ditchless systems. There were some similarities in species annotation between the two systems, with *Rhodoluna* being dominant in the ditched group, while ZOR0006 was dominant in the ditchless group, and *Candidatus* Bacilloplasma and norank_o__RsaHf231 had a larger proportion in both systems. There were significant differences in bacterial composition between samples of the same type from the two culture models (Figure 2B). *Anaerorhabdus furcosa*, *Exiguobacterium*, Clostridiaceae, and *Dysgonomonas* showed the most significant changes in both systems (Supplementary Figure 4C). In general, the alpha diversity of bacterial communities was similar between the two culture systems, but bacterial species composition differed according to the environment.

### Comparison of environmental microbiota among different rice-crayfish co-culture systems

**FIGURE 3:**
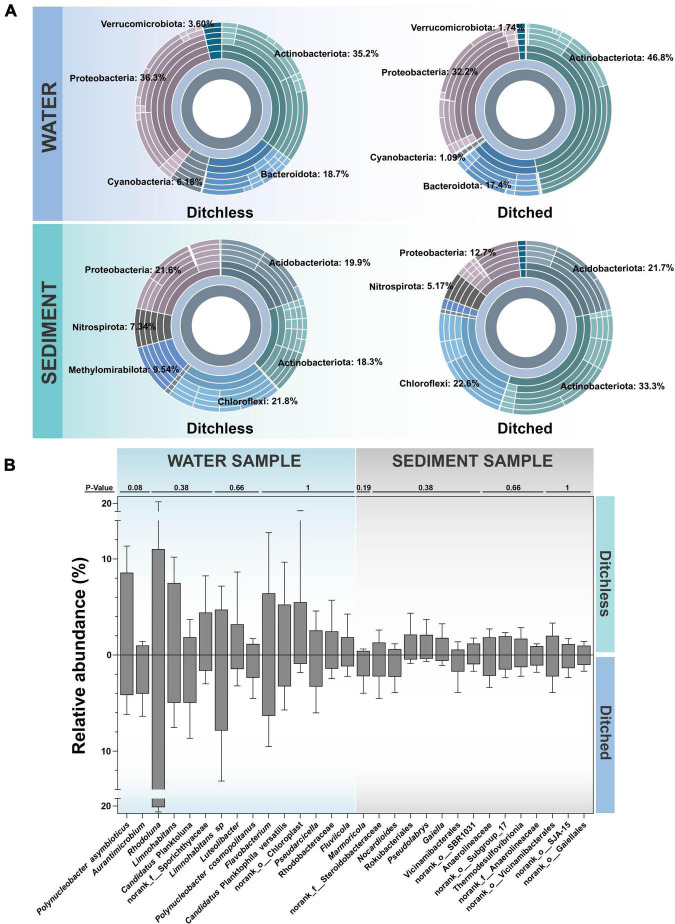
Bacterial community structures of the water and the sediment samples in the ditched and the ditchless systems. **(A)** Sunburst plots of the most abundant phyla. **(B)** The 15 most abundant OTUs. All data are expressed as the mean ± s.d. The difference test was completed by the Wilcoxon rank sum test.

Water and sediment samples from both systems were analyzed jointly to provide a broader picture of the differences in environmental bacterial communities. The sunburst plots in Figure 3A shows the overall bacterial communities at the phylum level in the water and sediment. Actinobacteriota, Proteobacteria and Bacteroidota had the highest abundance in the water. Notably, the abundance of Actinobacteriota was significantly higher in ditched water than in ditchless water samples, while Cyanobacteria and Verrucomicrobiota were more abundant in ditchless water, although their relative abundance was lower in the overall. Relative abundance of Methylomirabilota and Proteobacteria, as well as Actinobacteria, are clearly different in the sediments of the ditched and ditchless systems. On the other hand, similar to the composition of the water samples, Methylomirabilota and Nitrospirota, which had overall low relative abundance, were more abundant in the sediments of the ditchless group than in the ditched group. In addition, Actinobacteriota and Proteobacteria had higher abundances in both water and sediment and showed similar trends. Overall, the sediments exhibited a richer and more diverse bacterial community structure compared to the water samples. Although the two systems differed significantly in the bacterial composition of samples of the same type, the trends of variation were similar between the different types of samples.

At the species level, bacterial communities in water and sediment differed greatly between the ditched and ditchless systems. Further comparing the most abundant bacteria in the environment in the ditched and ditchless systems (Figure 3B), we found that the bacteria that exhibited the greatest differences in water were *Polynucleobacter asymbioticus* and *Aurantimicrobium*. *Polynucleobacter asymbioticus* was more abundant in the ditchless group, while *Aurantimicrobium* had a higher abundance in the ditched group. *Rhodoluna*, which had the greatest abundance in water, was more abundant in the ditched group. In the sediments, *Marmoricola* was most variable and more abundant in the ditch group, followed by Steroidobacteraceae and *Nocardioides* with similar distributions. Rokubacteriales, *Pseudolabrys*, and *Gaiella* show great differences in sediments and higher abundance in the ditchless group. Using FAPROTAX to predict the ecological function of sediment bacterial communities and select pathways related to ammonia nitrogen synthesis and utilization, given the enrichment and biochemical response capacity of sediments in aquaculture systems (Supplementary Figure 5). Microbiota in ditchless sediments acquire more mapping of nitrite respiration, sulfite respiration, chloroplasts, nitrogen fixation, and fermentation pathways. In contrast, ureolysis, nitrate respiration, nitrogen respiration, and nitrate reduction were more annotated in the ditched sediments.

### Lower abundance of potential pathogens in intestinal contents in ditchless systems

**FIGURE 4:**
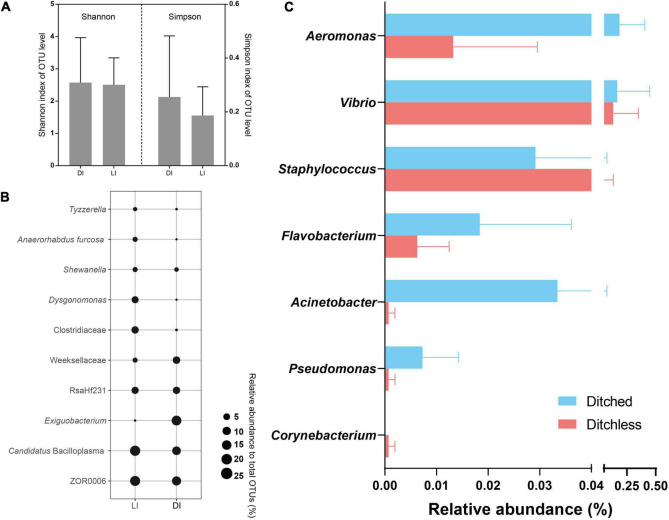
Bacterial community structures of crayfish intestine in the ditched and the ditchless systems. **(A)** Alpha diversity index (Shannon and Simpson) of OTUs. **(B)** Bubble plots of the 10 most abundant OTUs. **(C)** The relative abundance of common harmful bacteria. All data are expressed as the mean ± s.d.

The above studies show that bacterial communities in the environment were influenced by culture systems (geographical factors). To determine the effects of environmental changes on cultured species, bacterial communities in the crayfish intestine were compared. Although the Shannon and Simpson index changed between the two systems, they did not show significant (Figure 4A). We compared the relative abundance of the top 10 species in total abundance in intestinal contents. The results showed that there were some differences in the abundance of intestinal bacteria from different culture systems (Figure 4B). Specifically, the highest abundance of ZOR0006 and *Candidatus* Bacilloplasma were more predominant in the ditchless group. In addition, Clostridiaceae and *Dysgonomonas* also showed higher abundance in the ditchless group. In contrast, *Exiguobacterium* and Weeksellaceae were more abundant in the ditched group than in the ditchless group. Notably, *Exiguobacterium* was only annotated in DIF2, which resulted in a high standard deviation.

The abundant environmental bacterial community interfered with the performance of potential pathogens and reduced the signal-to-noise ratio of potential pathogens in the total community. To present the colonization patterns of potential pathogenic bacteria in the intestine more accurately, we selected seven categories of bacteria commonly infecting aquatic organisms and analyzed the differences in abundance under different culture systems (Figure 4C). The results showed that all these bacteria had colonized the intestine. The relative abundance of *Aeromonas*, *Vibrio*, *Flavobacterium*, *Acinetobacter*, and *Pseudomonas* was much lower in the intestine of ditchless cultured crayfish, while the relative abundance of *Staphylococcus* and *Corynebacterium* was higher, even though their absolute abundance was low. Interestingly, the relative abundance of these potentially pathogenic bacteria differed in sediment and water (Supplementary Figure 6). Briefly, the relative abundance of potential pathogenic bacteria in water were similar to those in the intestine, whereas the opposite pattern was observed in sediment.

### Intestinal microbiota of ditch-farmed crayfish are more susceptible to environmental microbiota

**FIGURE 5:**
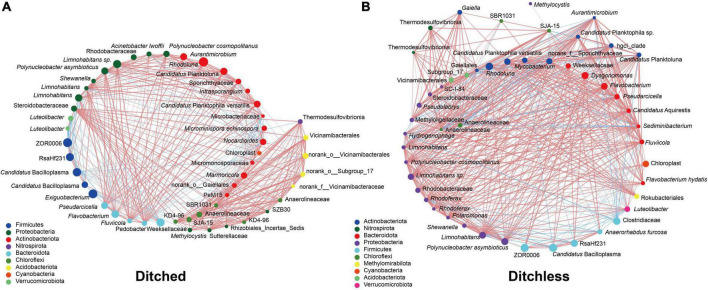
Combined analysis of bacterial community structures of water, sediment and intestine. Correlation network **(A,B)** of water, sediment and intestine in the ditched **(A)** and the ditchless **(B)** system. The size of the dots indicates the abundance of the most 50 OTUs. The red and blue lines indicate positive and negative correlations between the connecting points, respectively.

Despite the high degree of similarity between replicates of water, sediment, and gut contents (Supplementary Figures 2, 3), we sought to explore potential interactions between different sample types. Correlation networks were used to screen for dominant species in the total community. Determining the importance of species in the network based on Degree, Closeness, Betweenness centrality values. The results showed that norank_o__Gaiellales, norank_o__Subgroup_17, and *Nocardioides* were the dominant species in the bacterial community of the ditched group, while hgcI_clade, norank_f__Sporichthyaceae, and *Sediminibacterium* were dominant in the ditchless group (Figures 5A,B). Interestingly, the distribution of dominant species in water, sediment, and intestinal contents differed between culture systems. Specifically, the dominant species in the ditched group were distributed in water, sediment, and intestinal contents. In contrast, only norank_f__Sporichthyaceae in the ditchless group were distributed in all samples, and no *Sediminibacterium* and hgcI_clade were annotated in the intestinal samples, where hgcI_clade was annotated only in water. In addition, we did not find any significant functional differences between the bacterial communities of the ditched and ditchless systems (Supplementary Figures 7).

### Water depth and dissolved oxygen are the main environmental factors affecting the distribution of microbiota

**FIGURE 6:**
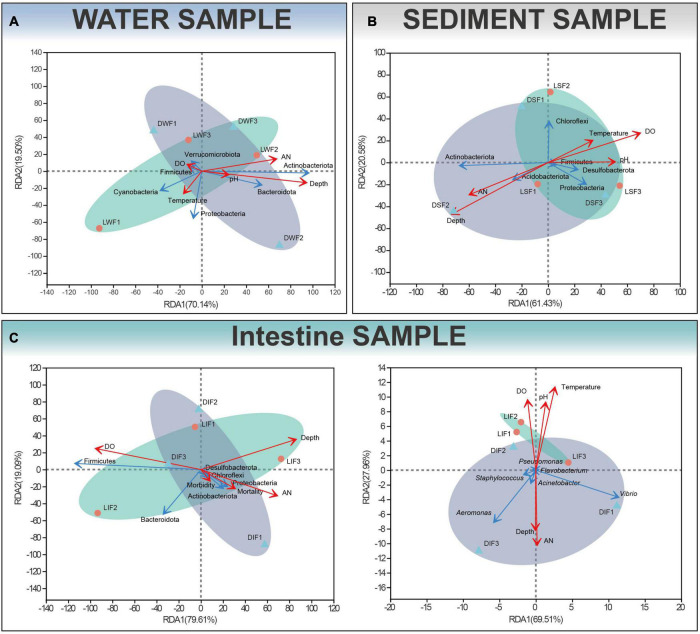
Redundancy analysis (RDA) of the relationship between bacterial and environmental factors in water **(A)**, sediment **(B)** and intestinal **(C)** samples for phylum levels and potentially pathogenic bacteria. Depth, AN and DO indicate water depth, total ammonia nitrogen and dissolved oxygen, respectively. Red and blue arrows represent environmental variables and bacteria respectively.

Microbiota of ditched and ditchless rice-crayfish co-culture systems showed significant differences by 16S rDNA sequencing analysis. Further, we would like to explain the reasons for the differences to apply to the actual farming process. Since the presence or absence of ditches is the largest perceptible change between the two farming systems, the resulting environmental changes were given priority consideration. RDA was used to assess the relationship between sample distribution, microbiota and environmental factors. The results showed that water depth was the decisive environmental factor affecting the distribution of water samples and was positively correlated with Actinobacteriota and Bacteroidota (Figure 6A). Water depth and dissolved oxygen dominate the distribution of sediment samples and are negatively correlated each other. Water depth was positively correlated with Actinobacteriota and Acidobacteriota, while dissolved oxygen was positively correlated with the combination of Firmicutes, Desulfobacterota, Proteobacteria, and Chloroflexi (Figure 6B). For the intestinal microbiota, dissolved oxygen is the most important factor affecting distribution, followed by water depth. In addition, morbidity and mortality were positively correlated with water depth and ammonia nitrogen, while negatively correlated with dissolved oxygen. Similarly, common pathogenic bacteria such as *Aeromonas* and *Vibrio* were positively correlated with water depth and ammonia nitrogen, while negatively correlated with the combination of dissolved oxygen, pH, and temperature (Figure 6C).

## Discussion

In this work, we tried to illustrate the potential microecological advantages of ditchless rice-crayfish co-culture systems by comparing the bacterial communities of conventional and ditchless rice-crayfish co-culture systems. The sequencing results showed that the environmental bacterial composition of the different systems was significantly different. Sediment bacteria from ditchless systems acquire less functional annotations related to ammonia nitrogen production. Relative abundance of pathogenic bacteria in the intestine of crayfish in ditchless systems is lower than in ditched systems. Water depth and dissolved oxygen are the main environmental factors that determine the distribution of microbiota.

Our sequencing results indicate that the two culture systems differ in the percentage of environmental bacteria at the phylum level. The alpha diversity of the bacterial community in the sediment was higher, compared to the potentially more fragile bacterial ecosystem in the water. This result is common in natural waters (Liao et al., 2020). In this study, the Actinobacteria was highly abundant in both water and sediment. This result is different from the results of some studies in which Proteobacteria were dominant in water (Wang et al., 2021). Water and sediment are in direct contact. Therefore, bacterial communities in both media may be more closely related in shallow water systems.

For cultured water, Cyanobacteria is also the dominant phylum, but it varies considerably in different systems. As photosynthetic autotrophic bacteria, this difference may be caused by the different light intensities received at different water depths (Haselkorn, 2009). Stronger light may also be responsible for higher dissolved oxygen in a ditchless environment (Andersen et al., 2017). In addition, dissolved oxygen usually shows a monotonic decrease along water depth. *Polynucleobacter asymbioticus* is the most diverse species-level bacteria in water. *Polynucleobacter asymbioticus* is widely distributed and is a prokaryotic prey for algal predation (Boenigk et al., 2004). Studies have shown that *Polynucleobacter asymbioticus* has different ecological adaptations and environmental preferences (Jezbera et al., 2011). The comparable abundance of *Polynucleobacter asymbioticus* in the two culture systems may laterally reflect the environmental differences. In addition, the abundance of *Polynucleobacter asymbioticus* was higher in the trenchless environment. This provides a more stable material basis for the energy flow of the ecological chain. *Aurantimicrobium* and *Rhodoluna* were shown to grow photoresponsive. This may account for the different abundance in different culture systems (Hempel et al., 2021).

For the bacterial community in the sediment, differences in the abundance of Actinobacteriota also reflect trends in the expression of Actinobacteriota in water. It has been reported that Actinobacteriota are sensitive to environmental conditions suitable for Cyanobacteria growth. This explains to some extent the unique bacterial community characteristics among different systems (Ghai et al., 2014). The large number of bacterial communities with unique metabolic functions in sediments is one of the main sources of ammonia nitrogen in aquaculture water environments (Zhu et al., 2019). Low relative abundance of different bacteria such as *Marmoricola*, Steroidobacteraceae, and *Nocardioides* in the sediment. However, bacterial communities in the ditchless sediments were less annotated for ammonia production and more annotated for ammonia consumption. Field tests also observed that ammonia nitrogen was significantly lower in the ditchless system than in the ditched system. Ammonia nitrogen is one of the major limiting factors for growth, survival and physiology in crustacean aquaculture. Excess ammonia nitrogen in the water and sediment can damage the aquatic ecosystem and induce disease (Chang et al., 2015). In addition, ammonia nitrogen causes a decrease in lymphocytes and phagocytes and suppression of immune responses in aquatic organisms. This may increase the risk of infection with pathogenic bacteria (Cheng and Chen, 2002). For benthic crayfish, ditchless culture environment that produces less ammonia nitrogen may be more conducive to maintaining the health of the organism and the balance of microecology.

In addition to the environmental microbiota, we also found some differences in the abundance of intestinal bacteria in different culture systems by analyzing the sequencing results. Except for ZOR0006, which was not mapped to any species, *Candidatus* Bacilloplasma showed high richness and comparability. Although studies have claimed that *Candidatus* Bacilloplasma is enriched in the intestine of crayfish with white feces syndrome, it has not been proven pathogenic (Hou et al., 2018). On the contrary, *Candidatus* Bacilloplasma, one of the only dominant genera in the crayfish intestine, is associated with crayfish physiological health (Huang et al., 2018). Higher abundance of potential pathogens in the intestinal contents of the ditched system explains the higher morbidity and mortality. Low dissolved oxygen in ditch systems may affect crayfish immune response and promote colonization of the intestine by potentially pathogenic bacteria (Le Moullac et al., 1998). Thus, compared to ditched systems, ditchless systems without digging ditches improve crayfish intestinal bacterial structure and facilitate crayfish survival. A close correlation between crayfish diseases and environmental microbiota has been observed (Xiong et al., 2014). Water quality degradation may have indirectly changed the crayfish intestine microbiota by altering the environmental bacterial community. However, the intestine microbiota should be more similar to the sediment bacterial community because pathogens in the environment can enter the intestine *via* the oral route (Soonthornchai et al., 2010; Xiong et al., 2017). However, the results of the present study were contrary to the speculation. A shorter breeding time may be a reasonable explanation for this anomaly. Since rice crayfish culture is concentrated in 3 months, sediments may not accumulate sufficiently. This leads to a greater impact of the culture water on the intestinal contents (Cornejo-Granados et al., 2017). As the main place for bacteria to live in the body, the intestine can form an effective barrier to isolate bacteria (Nakashima et al., 2018). However, key bacteria screened by the associated network were annotated in water, sediment and intestinal contents in the ditched system. This implies that the intestinal microbiota of crayfish in the ditched system interacted more strongly with the environmental microbiota than in the ditchless system. Combined with previous research results (Feng et al., 2021), crayfish cultured in ditched systems may be more susceptible to environmental microbiota. In addition, their intestinal structure may be more fragile and thus vulnerable to pathogenic bacteria.

Overall, bacterial communities in the ditchless model differed significantly from those in the traditional ditch model due to anthropogenic environmental alterations. Water depth and dissolved oxygen were the decisive environmental factors affecting bacterial structure. Compared to the traditional ditch model, the ditchless model showed microecological advantages, which were mainly reflected in ammonia nitrogen production and distribution of potentially pathogenic bacteria. Therefore, ditchless rice-crayfish farming is a worthy choice for environmentally friendly farming.

## Data Availability Statement

The data presented in this study are deposited in the Sequence Read Archive (SRA) at the NCBI repository, accession number: PRJNA816301.

## Ethics statement

All animal handling procedures were approved by the Animal Care and Use Committee of Sichuan Agricultural University (Chengdu, China), and followed the guidelines for animal experiments of Sichuan Agricultural University, under permit number: DY-2019202033.

## Author contributions

XH, ML, and YG conceived and designed the research. ML, YH, and HY performed the research and acquired the data. PO, DC, LY, and SY analyzed and interpreted the data. All authors were involved in drafting and revising the manuscript.

## Conflict of Interest

HY was employed by Haide Aquatic Technology Co., Ltd. The remaining authors declare that the research was conducted in the absence of any commercial or financial relationships that could be construed as a potential conflict of interest.

## Publisher’s Note

All claims expressed in this article are solely those of the authors and do not necessarily represent those of their affiliated organizations, or those of the publisher, the editors and the reviewers. Any product that may be evaluated in this article, or claim that may be made by its manufacturer, is not guaranteed or endorsed by the publisher.

## Funding

This research was supported by grants from the double support plan of Sichuan Agricultural University (No. 1921993230), Fund of Chengdu Science and Technology Bureau key Research and development support plan (No. 2022-YF05-00636-SN), Sichuan Science and technology plan project (No. 2022NZZJ0014), and Opening Fund of Key Laboratory of Sichuan Province for Fishes Conservation and Utilization in the Upper Reaches of the Yangtze River (NJTCSC09).

## Supplementary Material

The Supplementary Material for this article can be found online at: https://www.frontiersin.org/articles/10.3389/fmicb.2022.892026/full#supplementary-material

## Footnotes

1
http://drive5.com/uparse

2
https://cloud.majorbio.com/

## References

[B1] Affairs (2021). *Technical Specifications for Rice and Fishery Farming Riceshrimp*. DB51/T 2754-2021. Administration for Market Regulation of Sichuan Province.

[B2] Andersen M. R. Kragh T. Sand-Jensen K. (2017). Extreme diel dissolved oxygen and carbon cycles in shallow vegetated lakes. *Proc. Biol. Sci.* 284:20171427. 10.1098/rspb.2017.1427 PMC5597838 28904141

[B3] Boenigk J. Stadler P. Wiedlroither A. Hahn M. W. (2004). Strain-specific differences in the grazing sensitivities of closely related ultramicrobacteria affiliated with the Polynucleobacter cluster. *Appl. Environ. Microbiol.* 70 5787–5793. 10.1128/aem.70.10.5787-5793.2004 15466515 PMC522116

[B4] Cao C. Jiang Y. Wang J. Yuan P. Chen S. (2017). Dual character” of rice-crayfish culture and strategy for its sustainable development. *Chin. J. Eco-Agric.* 25 1245–1253. 10.13930/j.cnki.cjea.170739

[B5] Cerenius L. Jiravanichpaisal P. Liu H. P. Söderhill I. (2010). Crustacean immunity. *Adv. Exp. Med. Biol.* 708 239–259. 10.1007/978-1-4419-8059-5_13 21528702

[B6] Chang Z. W. Chiang P. C. Cheng W. Chang C. C. (2015). Impact of ammonia exposure on coagulation in white shrimp, Litopenaeus vannamei. *Ecotoxicol. Environ. Saf.* 118 98–102. 10.1016/j.ecoenv.2015.04.019 25916769

[B7] Chen X. Fan L. Qiu L. Dong X. Wang Q. Hu G. (2021). Metagenomics Analysis Reveals Compositional and Functional Differences in the Gut Microbiota of Red Swamp Crayfish, *Procambarus clarkii*, Grown on Two Different Culture Environments. *Front. Microbiol.* 12:735190. 10.3389/fmicb.2021.735190 PMC8558459 34733252

[B8] Cheng W. Chen J. C. (2002). The virulence of Enterococcus to freshwater prawn *Macrobrachium rosenbergii* and its immune resistance under ammonia stress. *Fish Shellfish Immunol.* 12 97–109. 10.1006/fsim.2001.0363 11911679

[B9] Cornejo-Granados F. Lopez-Zavala A. A. Gallardo-Becerra L. Mendoza-Vargas A. Sánchez F. Vichido R. (2017). Microbiome of Pacific Whiteleg shrimp reveals differential bacterial community composition between Wild, Aquacultured and AHPND/EMS outbreak conditions. *Sci. Rep.* 7:11783. 10.1038/s41598-017-11805-w PMC5603525 28924190

[B10] Feng Y. Li M. Duan H. Li L. Ouyang P. Chen D. (2021). Microbial analysis reveals the potential colonization of pathogens in the intestine of crayfish (*Procambarus clarkii*) in traditional aquaculture environments. *Ecotoxicol. Environ. Saf.* 224:112705. 10.1016/j.ecoenv.2021.112705 34454354

[B11] Ghai R. Mizuno C. M. Picazo A. Camacho A. Rodriguez-Valera F. (2014). Key roles for freshwater Actinobacteria revealed by deep metagenomic sequencing. *Mol. Ecol.* 23 6073–6090. 10.1111/mec.12985 25355242

[B12] Haselkorn R. (2009). Cyanobacteria. *Curr. Biol.* 19 R277–R278. 10.1016/j.cub.2009.01.016 19368866

[B13] Hempel P. P. Keffer J. L. Maresca J. A. (2021). RNA-Seq Reveals that Light and Darkness Are Different Stimuli in Freshwater Heterotrophic Actinobacteria. *Front. Microbiol.* 12:739005. 10.3389/fmicb.2021.739005 PMC8591293 34790178

[B14] Hou D. Huang Z. Zeng S. Liu J. Wei D. Deng X. (2018). Intestinal bacterial signatures of white feces syndrome in shrimp. *Appl. Microbiol. Biotechnol.* 102 3701–3709. 10.1007/s00253-018-8855-2 29516144

[B15] Hou D. Zhou R. Zeng S. Wei D. Deng X. Xing C. (2020). Intestine Bacterial Community Composition of Shrimp Varies Under Low- and High-Salinity Culture Conditions. *Front. Microbiol.* 11:589164. 10.3389/fmicb.2020.589164 PMC7701045 33304335

[B16] Hou J. Zhang D. Zhu J. (2021). Nutrient accumulation from excessive nutrient surplus caused by shifting from rice monoculture to rice-crayfish rotation. *Environ. Pollut.* 271:116367. 10.1016/j.envpol.2020.116367 33418287

[B17] Hu L. Zhang J. Ren W. Guo L. Cheng Y. Li J. (2016). Can the co-cultivation of rice and fish help sustain rice production? *Sci. Rep.* 6:28728. 10.1038/srep28728 PMC4923892 27349875

[B18] Huang F. Pan L. Song M. Tian C. Gao S. (2018). Microbiota assemblages of water, sediment, and intestine and their associations with environmental factors and shrimp physiological health. *Appl. Microbiol. Biotechnol.* 102 8585–8598. 10.1007/s00253-018-9229-5 30039332

[B19] Huang J. Xu X. Zhou Z. Deng Y. Hu H. Huang B. (2020). Crayfish farming technology in rice fields without ring ditches. *Jiangxi Fish. Sci. Technol.* 02:31–32.

[B20] Huang Z. Zeng S. Xiong J. Hou D. Zhou R. Xing C. (2020). Microecological Koch’s postulates reveal that intestinal microbiota dysbiosis contributes to shrimp white feces syndrome. *Microbiome* 8:32. 10.1186/s40168-020-00802-3 PMC7065354 32156316

[B21] Jezbera J. Jezberová J. Brandt U. Hahn M. W. (2011). Ubiquity of Polynucleobacter necessarius subspecies asymbioticus results from ecological diversification. *Environ. Microbiol.* 13 922–931. 10.1111/j.1462-2920.2010.02396.x 21208356 PMC3087241

[B22] Le Moullac G. Soyez C. Saulnier D. Ansquer D. Avarre J. C. Levy P. (1998). Effect of hypoxic stress on the immune response and the resistance to vibriosis of the shrimpPenaeus stylirostris. *Fish Shellfish Immunol.* 8 621–629. 10.1006/fsim.1998.0166

[B23] Li Y. Guo X. Cao X. Deng W. Luo W. Wang W. (2012). Population genetic structure and post-establishment dispersal patterns of the red swamp crayfish *Procambarus clarkii* in China. *PLoS One* 7:e40652. 10.1371/journal.pone.0040652 PMC3393698 22808222

[B24] Liao H. Yen J. Y. Guan Y. Ke D. Liu C. (2020). Differential responses of stream water and bed sediment microbial communities to watershed degradation. *Environ. Int.* 134:105198. 10.1016/j.envint.2019.105198 31704564

[B25] MARA (2021). 2021 Report on the development of crayfish industry in China. *China Fish* 07:27–33.

[B26] Mueller K. Ash C. Pennisi E. Smith O. (2012). The gut microbiota. Introduction. *Science* 336:1245. 10.1126/science.336.6086.1245 22674336

[B27] Nakashima K. Kimura S. Ogawa Y. Watanabe S. Soma S. Kaneko T. (2018). Chitin-based barrier immunity and its loss predated mucus-colonization by indigenous gut microbiota. *Nat. Commun.* 9:3402. 10.1038/s41467-018-05884-0 PMC6109156 30143642

[B28] Shen G. Zhang X. Gong J. Wang Y. Huang P. Shui Y. (2020). Transcriptomic analysis of *Procambarus clarkii* affected by “Black May” disease. *Sci. Rep.* 10:21225. 10.1038/s41598-020-78191-8 PMC7719172 33277587

[B29] Si G. Peng C. Yuan J. Xu X. Zhao S. Xu D. (2017). Changes in soil microbial community composition and organic carbon fractions in an integrated rice-crayfish farming system in subtropical China. *Sci. Rep.* 7:2856. 10.1038/s41598-017-02984-7 PMC5460161 28588212

[B30] Soonthornchai W. Rungrassamee W. Karoonuthaisiri N. Jarayabhand P. Klinbunga S. Söderhäll K. (2010). Expression of immune-related genes in the digestive organ of shrimp, Penaeus monodon, after an oral infection by Vibrio harveyi. *Dev. Comp. Immunol.* 34 19–28. 10.1016/j.dci.2009.07.007 19646472

[B31] Wang Y. Wang C. Chen Y. Zhang D. Zhao M. Li H. (2021). Microbiome Analysis Reveals Microecological Balance in the Emerging Rice-Crayfish Integrated Breeding Mode. *Front. Microbiol.* 12:669570. 10.3389/fmicb.2021.669570 PMC8219076 34168630

[B32] Xiong J. Zhu J. Zhang D. (2014). The application of bacterial indicator phylotypes to predict shrimp health status. *Appl. Microbiol. Biotechnol.* 98 8291–8299. 10.1007/s00253-014-5941-y 25042597

[B33] Xiong J. Zhu J. Dai W. Dong C. Qiu Q. Li C. (2017). Integrating gut microbiota immaturity and disease-discriminatory taxa to diagnose the initiation and severity of shrimp disease. *Environ. Microbiol.* 19 1490–1501. 10.1111/1462-2920.13701 28205371

[B34] Zhao Z. Jiang J. Pan Y. Dong Y. Chen Z. Zhang G. (2020). Temporal dynamics of bacterial communities in the water and sediments of sea cucumber (Apostichopus japonicus) culture ponds. *Aquaculture* 528:735498. 10.1016/j.aquaculture.2020.735498

[B35] Zhu Y. Jin X. Tang W. Meng X. Shan B. (2019). Comprehensive analysis of nitrogen distributions and ammonia nitrogen release fluxes in the sediments of Baiyangdian Lake. *China J. Environ. Sci.* 76 319–328. 10.1016/j.jes.2018.05.024 30528023

