# Supplementary material for: Microbiome analysis reveals microecological advantages of emerging ditchless rice-crayfish co-culture mode
Source: Front Microbiol. 2022 Jul 22;13:892026. doi: 10.3389/fmicb.2022.892026 (PMC9355531; doi:10.3389/fmicb.2022.892026)
Supplement: Supplementary file 1 [file Data_Sheet_1.docx]

**Supplementary materials**

**
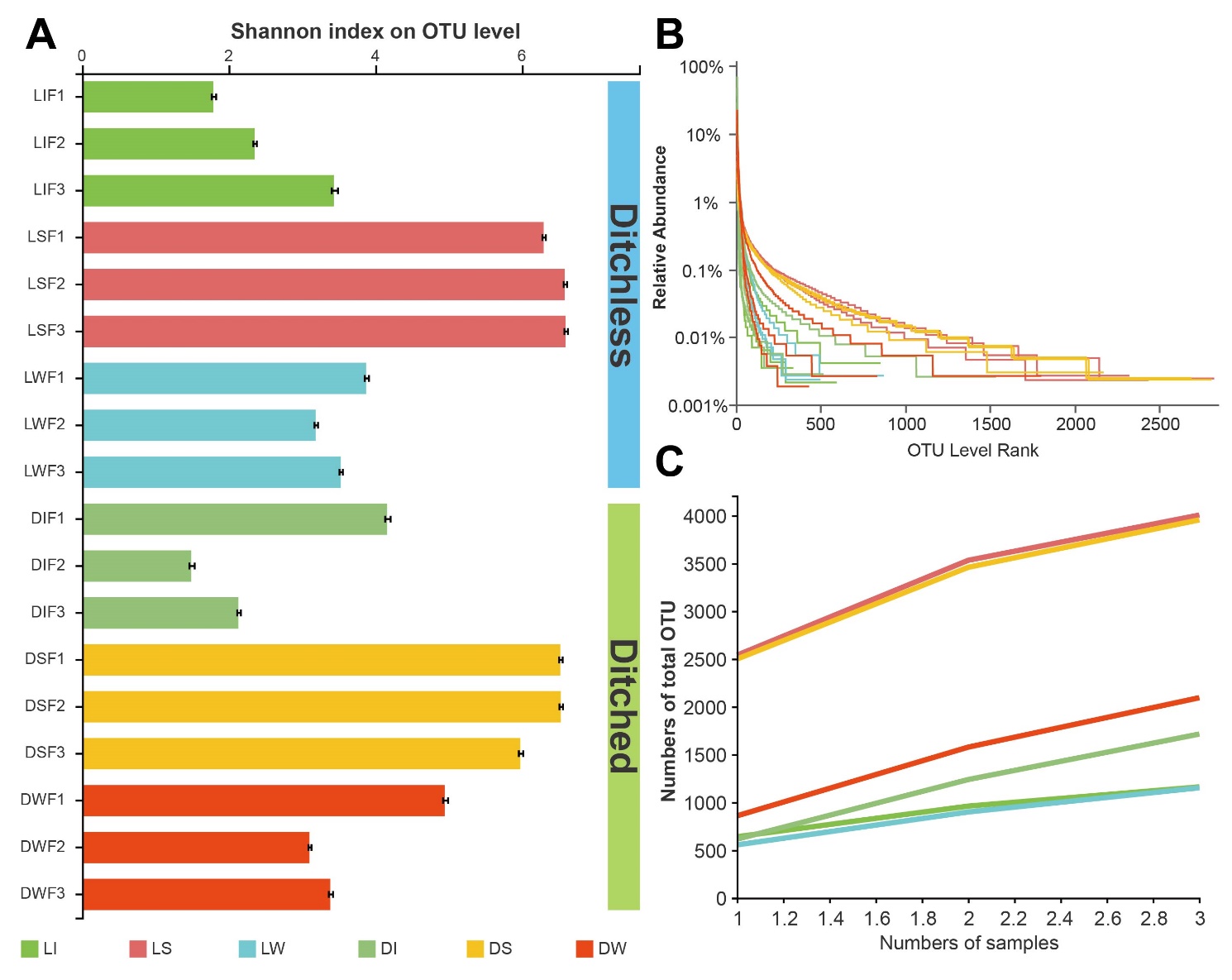
**

**Fig. S1**. **Species annotation and assessment**. A: Shannon index of each replicate. B: Rank-abundance curve of samples. The x-axis represents the ranking level of the number of OTUs at the taxonomic level, and the y-axis represents the relative percentage of the number of species. C: Dilution curves of samples at OTU levels.**
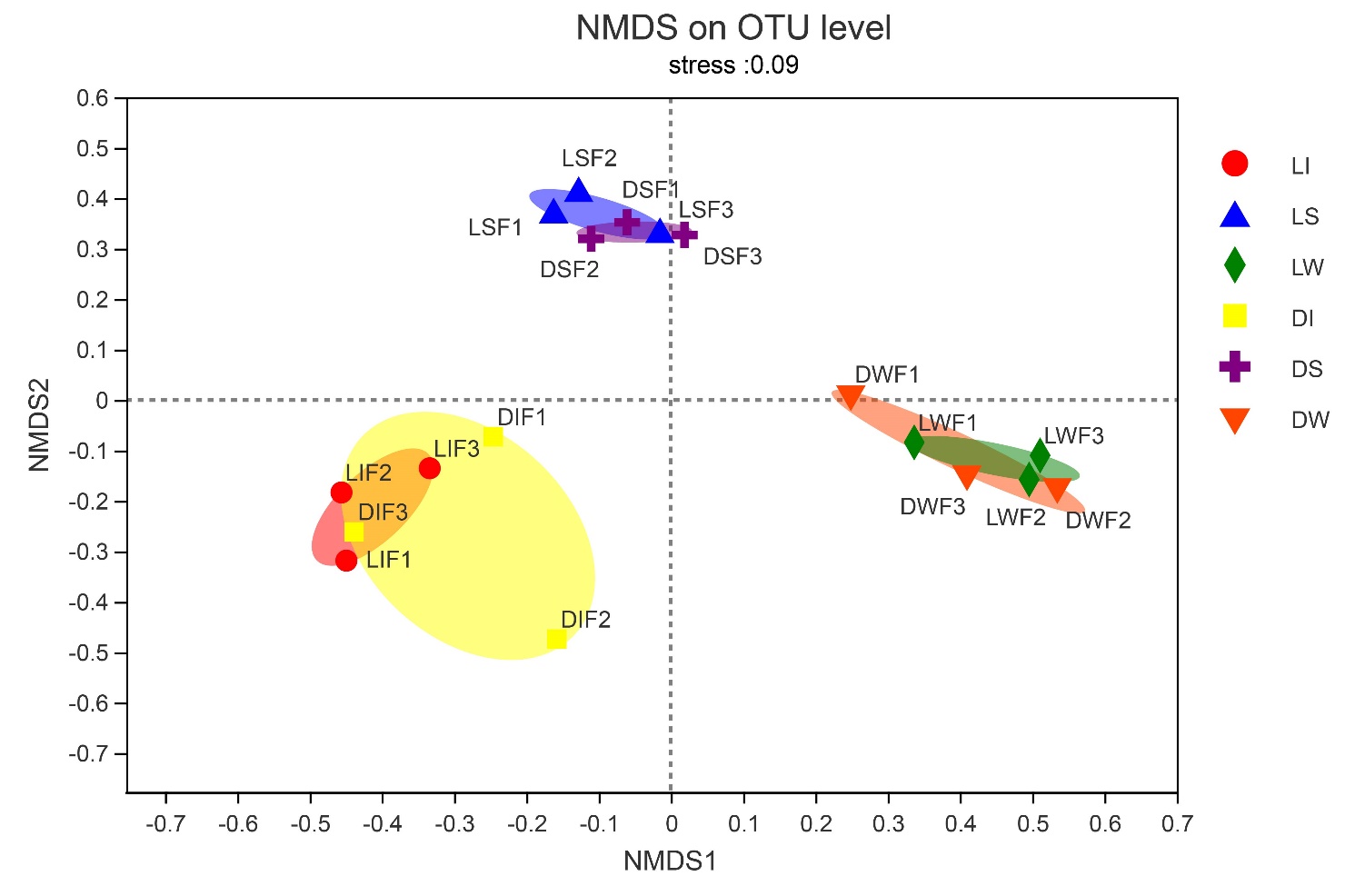
**

**Fig. S2. Non-metric multidimensional scale analysis (NMDS) of bacterial communities at the operational taxonomic unit (OTU) level.**

**
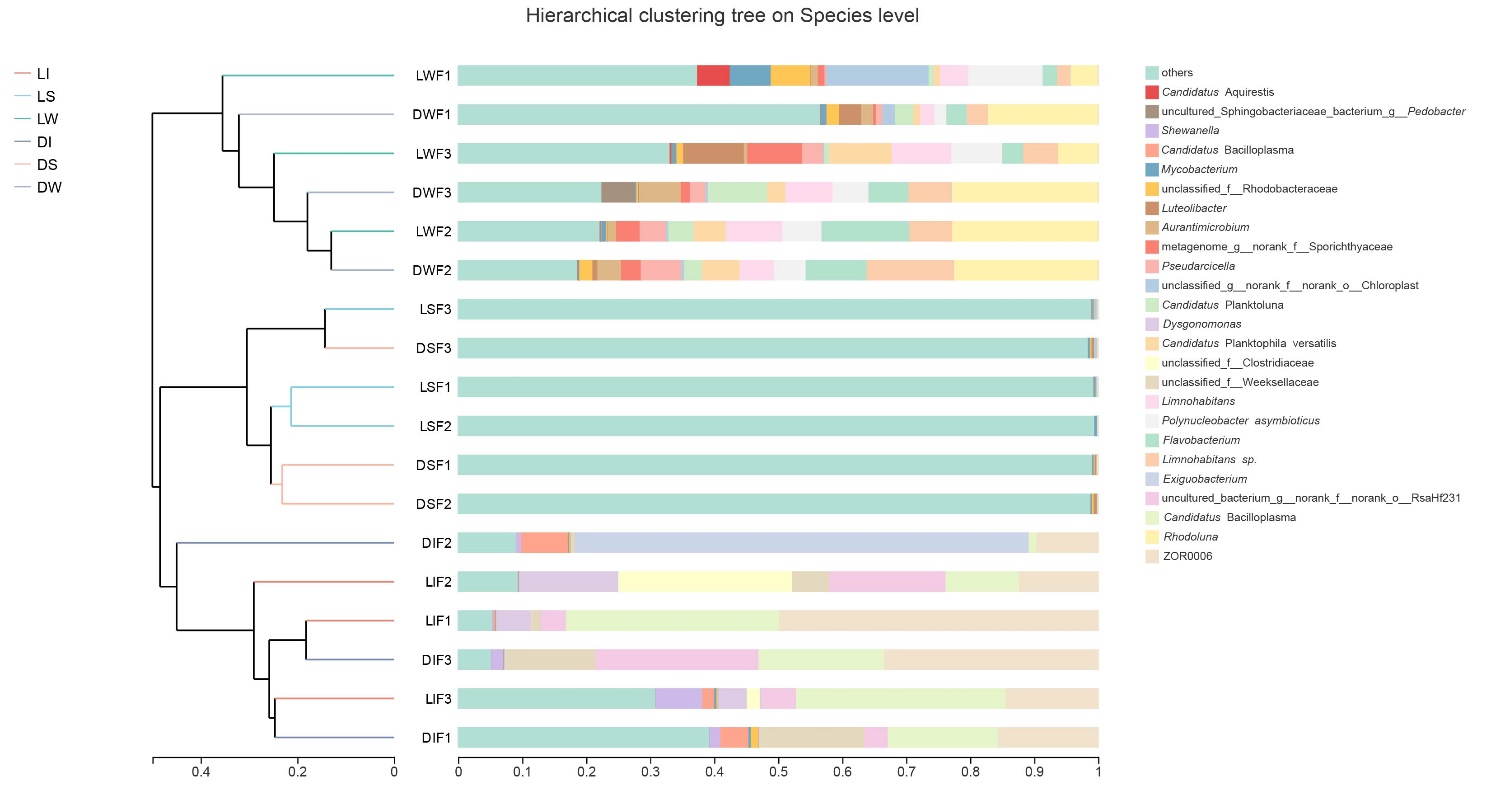
**

**Fig. S3. Hierarchical clustering analysis based on OTU at the species level.**

**
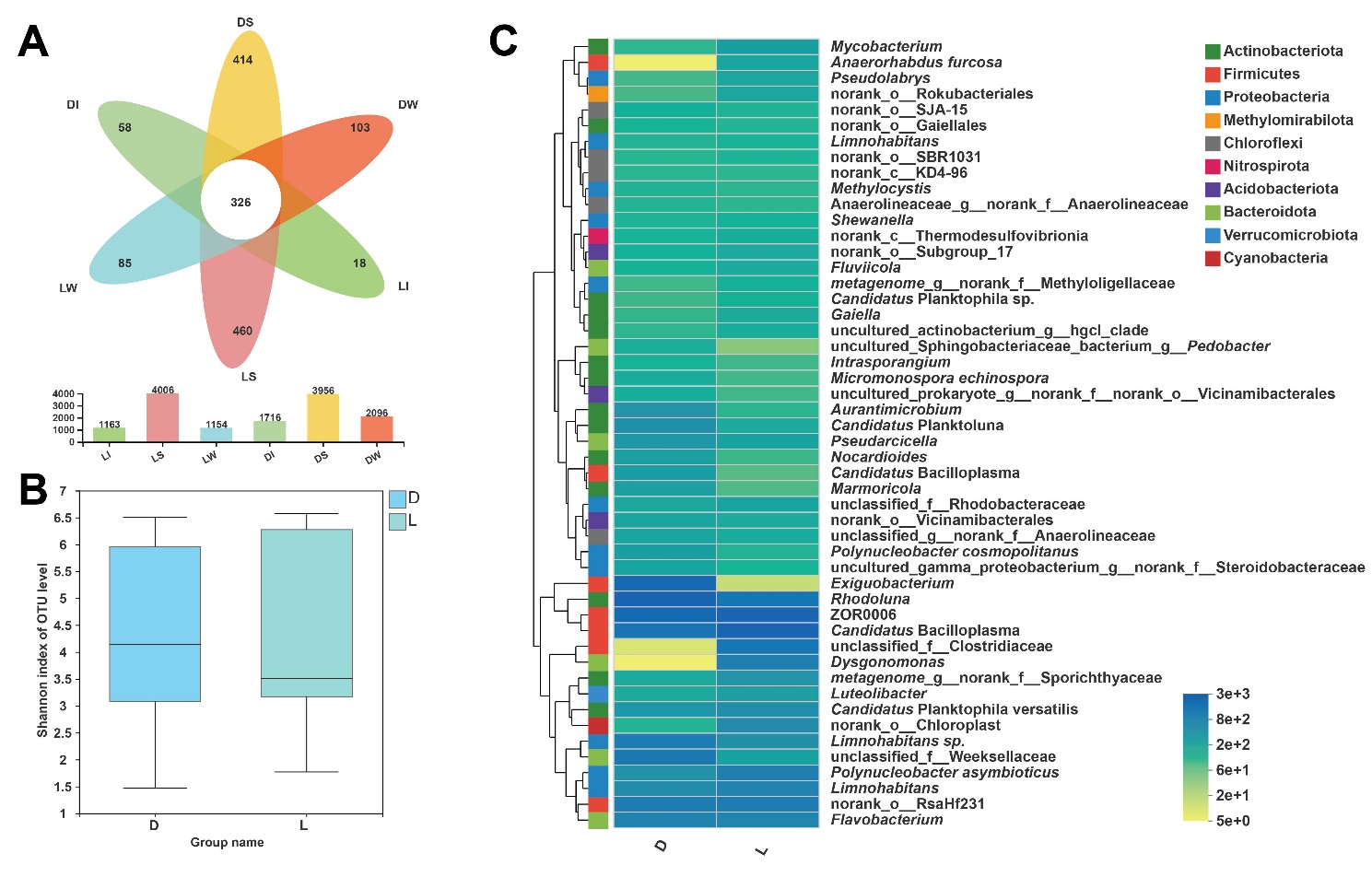
**

**Fig. S4. Comparison of microbial community richness and diversity between ditched and ditchless samples.** A: Venn diagram of the number of bacterial species annotated at the OTU level for different types of samples. B: Comparison of the Shannon index at the OTU level between the two systems. C: Heat map of microbial community composition at the species level for two systems.

**
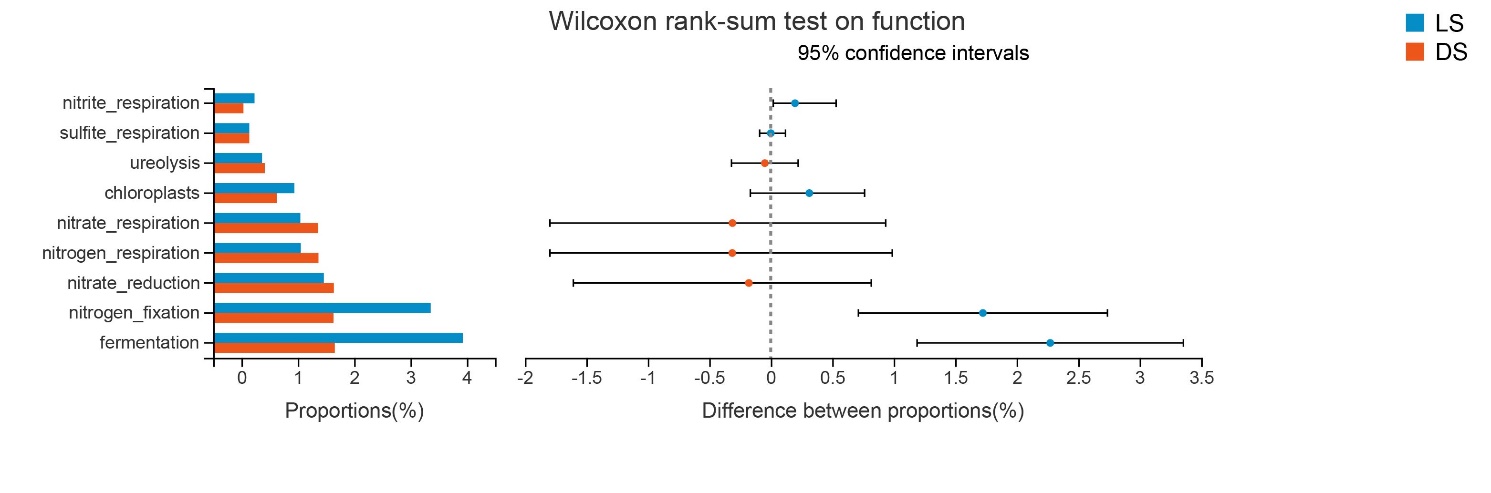
**

**Fig. S5. Prediction of FAPROTAX function in sediment microbial communities.** Wilcoxon rank sum test was used for the difference test with multiple corrections using Bonferroni.


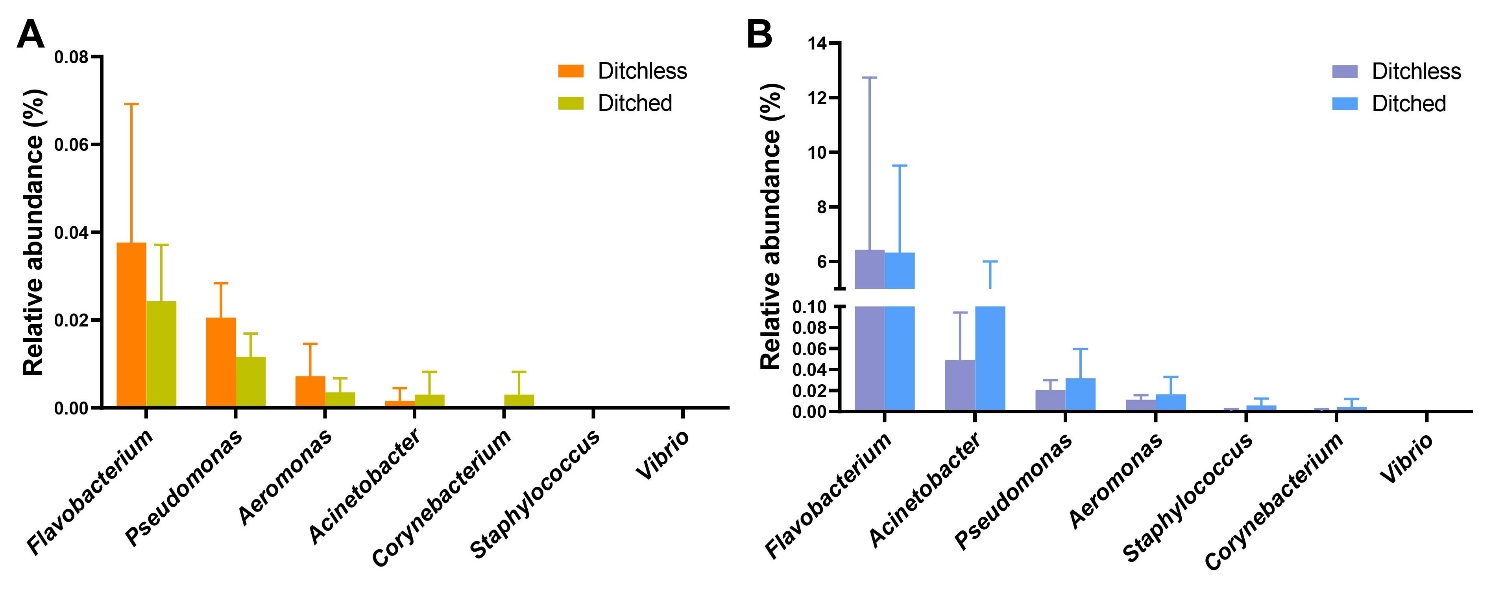


**Fig. S6. Comparison of common harmful bacteria in environmental samples.** The chart lists the relative abundance of common harmful bacteria in sediment (A) and water (B), and the data are expressed as mean ± standard deviation.

**
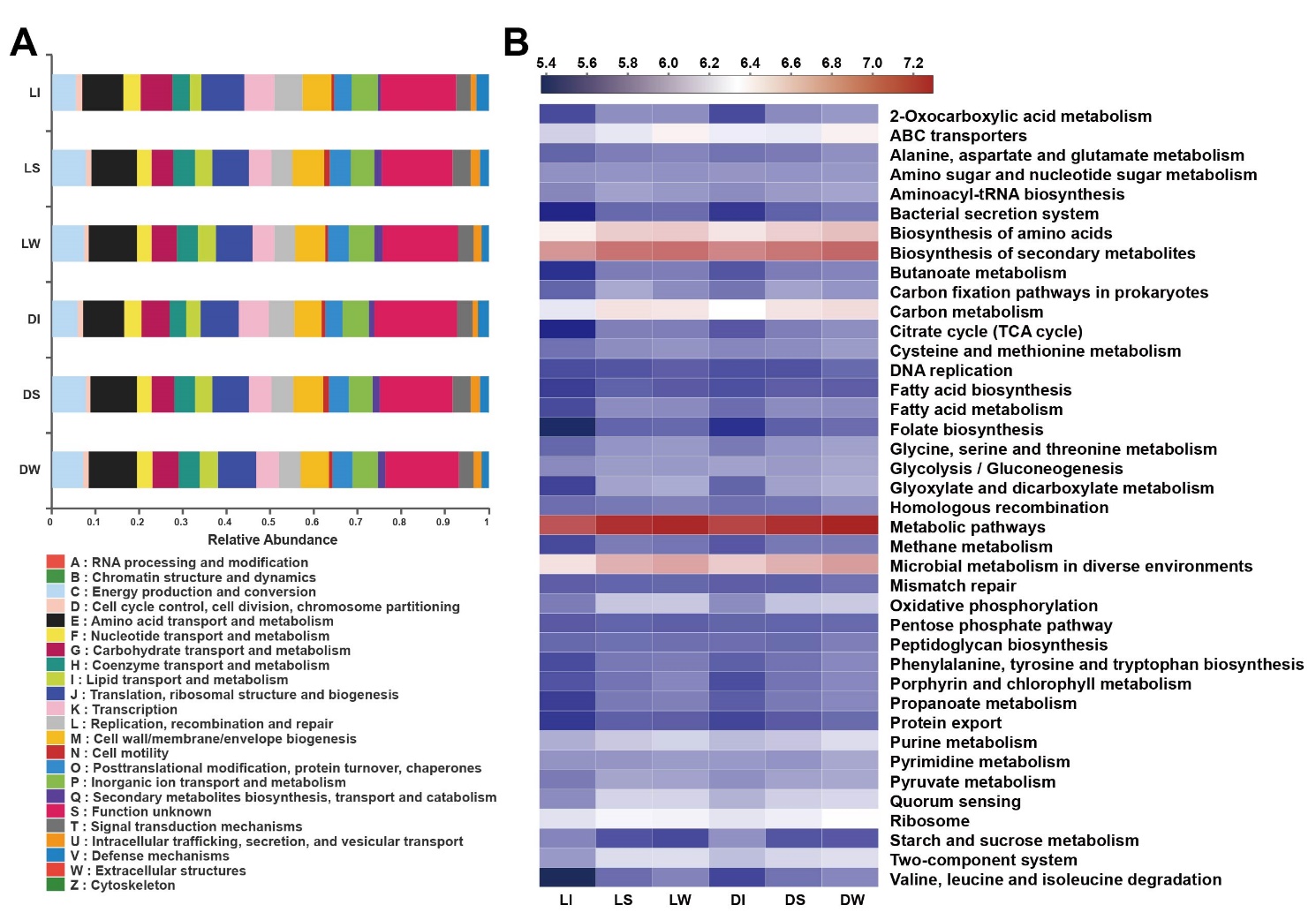
**

**Fig. S7**. **Functional prediction of microbial communities**. A: COG prediction for water, sediment, and intestinal contents samples from the ditchless and ditched systems. B: KEGG thermograms of water, sediment, and intestinal contents samples from the ditchless and ditched systems.

**Table S1**

Sequencing characteristics and diversity of microbial communities

| Mode | Sample type | Category | Replicate | Seq_num | Base_num | Mean_length | Min_length | Max_length | Shannon | Simpson | Chao | Coverage |
| --- | --- | --- | --- | --- | --- | --- | --- | --- | --- | --- | --- | --- |
| Ditched | INTESTINE | DI | F1 | 40,249 | 16,944,675 | 421.00 | 388 | 501 | 4.14 | 0.07 | 1896.51 | 0.987746 |
|  |  |  | F2 | 30,748 | 13,129,242 | 426.99 | 367 | 431 | 1.47 | 0.51 | 424.53 | 0.996247 |
|  |  |  | F3 | 36,807 | 15,694,774 | 426.41 | 337 | 434 | 2.11 | 0.19 | 828.97 | 0.993128 |
|  | SEDIMENT | DS | F1 | 61,869 | 25,762,439 | 416.40 | 223 | 509 | 6.50 | 0.01 | 3398.07 | 0.982928 |
|  |  |  | F2 | 53,893 | 22,447,207 | 416.51 | 219 | 458 | 6.51 | 0.00 | 3121.95 | 0.984505 |
|  |  |  | F3 | 43,027 | 18,058,750 | 419.71 | 311 | 479 | 5.96 | 0.01 | 2822.55 | 0.979258 |
|  | WATER | DW | F1 | 43,569 | 18,146,890 | 416.51 | 229 | 448 | 4.93 | 0.04 | 2474.96 | 0.982917 |
|  |  |  | F2 | 65,434 | 27,387,656 | 418.55 | 345 | 458 | 3.08 | 0.09 | 697.73 | 0.996503 |
|  |  |  | F3 | 41,832 | 17,454,637 | 417.26 | 311 | 477 | 3.37 | 0.08 | 1322.93 | 0.989721 |
| Ditchless | INTESTINE | LI | F1 | 32,850 | 14,027,533 | 427.02 | 359 | 444 | 1.77 | 0.31 | 644.68 | 0.993392 |
|  |  |  | F2 | 49,784 | 20,896,599 | 419.75 | 337 | 431 | 2.33 | 0.15 | 1092.78 | 0.993532 |
|  |  |  | F3 | 32,935 | 13,938,292 | 423.21 | 337 | 496 | 3.41 | 0.10 | 1325.01 | 0.985195 |
|  | SEDIMENT | LS | F1 | 60,909 | 25,506,237 | 418.76 | 255 | 504 | 6.28 | 0.01 | 3188.92 | 0.983061 |
|  |  |  | F2 | 57,391 | 23,921,002 | 416.81 | 271 | 451 | 6.56 | 0.00 | 2788.84 | 0.985257 |
|  |  |  | F3 | 51,488 | 21,598,356 | 419.48 | 246 | 509 | 6.58 | 0.00 | 3301.96 | 0.983291 |
|  | WATER | LW | F1 | 49,039 | 20,495,290 | 417.94 | 212 | 472 | 3.85 | 0.05 | 1374.22 | 0.989708 |
|  |  |  | F2 | 47,777 | 19,984,633 | 418.29 | 311 | 458 | 3.17 | 0.09 | 768.13 | 0.99518 |
|  |  |  | F3 | 46,907 | 19,689,833 | 419.76 | 322 | 431 | 3.51 | 0.05 | 805.44 | 0.994492 |

**Table S2**

Physical and chemical properties of sampling sites and culture conditions

| Data category | Index | Unit | Ditched culture | Ditchless culture | P value | Significance |
| --- | --- | --- | --- | --- | --- | --- |
| Water Quality | Water depth | cm | 76.7±2.9 | 63.3±1.5 | 2.11E-03 | ** |
|  | Maximum depth | cm | 181.3±4.7 | 82.3±5.5 | 1.90E-05 | *** |
|  | Minimum depth | cm | 54.7±2.1 | 51.3±1.2 | 0.07 |  |
|  | Temperature | ℃ | 18.9±0.10 | 19.2±0.10 | 0.02 | * |
|  | pH |  | 8.13±0.06 | 8.30±0.10 | 0.07 |  |
|  | Dissolved oxygen | mg/L | 6.07±0.06 | 6.53±1.12 | 3.32E-03 | ** |
|  | Ammonia nitrogen | mg/L | 0.12±0.01 | 0.01±0.01 | 1.57E-04 | *** |
|  | Nitrite | mg/L | 0.06±0.01 | 0.01±0.01 | 2.19E-03 | ** |
|  | Transparency | m | 0.32±0.01 | 0.31±0.01 | 0.37 |  |
| Culture status | Morbidity | % | 17.67±0.58 | 4.67±1.53 | 1.60E-04 | *** |
|  | Mortality | % | 13.00±1.00 | 1.33±0.58 | 6.30E-05 | *** |
|  | Growth rate | % | 3.03±0.21 | 3.20±0.10 | 0.28 |  |
|  | Sales price | ¥ / 500g | 25 | 27 | N/A | NA |

^*^, ^**^ and ^***^ represent *P* < 0.05, *P* < 0.01 and *P* < 0.001, respectively.
